# Structural Stigma and Sexual Minority Victimization Across 28 Countries: The Moderating Role of Gender, Gender Nonconformity, and Socioeconomic Status

**DOI:** 10.1177/08862605221108087

**Published:** 2022-08-08

**Authors:** Richard Bränström, Daniel Fellman, John Pachankis

**Affiliations:** 1 Department of Clinical Neuroscience, Karolinska Institute, Stockholm, Sweden; 2 Department of Social and Behavioral Sciences, Yale School of Public Health, New Haven, CT, USA

**Keywords:** lesbian, gay, bisexual, stigma, prejudice, discrimination, mental health

## Abstract

**Objective:**

Country-level structural stigma toward sexual minority individuals (i.e., discriminatory laws and policies and prejudicial attitudes) shows robust associations with sexual minority individuals’ mental health and individual-level stigma processes, such as identity concealment. Whether structural stigma is also associated with interpersonal-level stigma processes, such as victimization, is rarely studied. Whether the association between structural stigma and sexual minority individuals’ interpersonal mistreatment varies across gender, gender nonconformity, and socioeconomic status also remains to be determined.

**Methods:**

In 2012, sexual minority adults (*n* = 86,308) living in 28 European countries responded to questions assessing past-12-month victimization experiences (i.e., physical or sexual attack or threat of violence). Country-level structural stigma was objectively indexed as an aggregate of national laws, policies, and population attitudes negatively affecting sexual minority individuals

**Results:**

Country-level structural stigma was significantly associated with victimization (adjusted odds ratios [AOR]: 1.13, 95% confidence interval [CI]: 1.04–1.22; *p* = .004). However, this effect varied by gender, gender nonconformity, and socioeconomic status. For both sexual minority men and women, gender nonconformity and lower socioeconomic status were associated with increased risk of victimization. The strongest association between country-level stigma and victimization was found among gender nonconforming men with lower socioeconomic status (AOR: 1.32, 95% CI: 1.14–1.52; *p* < .001).

**Conclusions:**

A much larger proportion of sexual minorities living in higher stigma countries reports victimization than those living in lower stigma countries. At the same time, the association between country-level structural stigma and victimization is most heavily concentrated among gender nonconforming men with lower socioeconomic status.

## Introduction

Sexual minority individuals (e.g., those who identify as lesbian, gay, bisexual, and pansexual) experience a significantly elevated risk of diminished health and well-being compared to heterosexual individuals (King et al., 2008; Patterson et al., 2020; Plöderl & Tremblay, 2015). The source of this disparity can be found in sexual minority individuals’ disproportionate experience of identity-related stress at multiple levels (Hatzenbuehler et al., 2010; Maiolatesi et al., 2021; Mays & Cochran, 2001). Structural stigma represents the most pervasive level of identity-related stress surrounding sexual minority individuals and is defined as laws and policies that deny, or fail to protect, the equal rights of sexual minority individuals, as well as prejudicial population attitudes (Hatzenbuehler, 2016). Research into structural stigma and its associations with mental health typically creates an index of structural stigma at the level of country (Pachankis & Bränström, 2018; Pachankis et al., 2021), U.S. state (Hatzenbuehler et al., 2010), or more local municipality (Lattanner et al., 2021) and links this index to measures of mental health among the sexual minorities living in those geographic units. This research typically finds that country-level (Pachankis & Bränström, 2018; Pachankis et al., 2021) and U.S. state-level (Hatzenbuehler, 2011) structural stigma is robustly associated with sexual minority individuals’ poor mental health.

Although a relatively large body of research demonstrates associations between structural stigma and poor mental health (Hatzenbuehler, 2016), more recent research has sought to identify the mechanisms through which structural stigma might operate to exert an adverse impact on mental health (Hatzenbuehler et al., 2018; Lattanner et al., 2021; Pachankis et al., 2021; van der Star et al., 2021). This research has mostly focused on individual-level psychological reactions to stigma that might serve as mechanisms linking structural stigma to poor mental health. For example, studies have found that structural stigma toward sexual minority individuals is associated with sexual orientation identity concealment when structural stigma is measured at the level of European country (Pachankis & Bränström, 2018) and U.S. state and local municipality (Lattanner et al., 2021). Other research has shown country-level structural stigma to be related to sexual minority men’s internalization of stigma (Pachankis et al., 2021), another psychological processes known to be associated with mental health (Newcomb & Mustanski, 2010).

While these studies highlight associations between structural stigma and individual-level psychological processes, such as identity concealment and internalized stigma, few studies have examined associations between structural stigma and interpersonal manifestations of stigma. Those that have examined interpersonal correlates of structural stigma have tended to focus on bullying among sexual minority youth (Hatzenbuehler & Keyes, 2013; Meyer et al., 2019; van der Star et al., 2021). For instance, in U.S. states that do not enumerate sexual orientation in legal protections against bullying, sexual minority youth living in those states report greater perceived lack of safety at school and an increased risk of suicide attempts (Meyer et al., 2019). Indeed, structural stigma might provide cover for, or justify failing to mete out punishment against, individuals living in structurally stigmatizing geographies who engage in interpersonal attacks against sexual minority individuals. In adulthood, sexual minority individuals have a higher risk of being exposed to victimization, including physical and sexual assaults and threats of violence, compared to heterosexual individuals (Flores et al., 2020; Friedman et al., 2011). Yet, to our knowledge, only one study has examined adulthood victimization as a function of structural stigma (van der Star et al., 2021) and found that victimization both in early life and during adulthood partially explained variations in life satisfaction among sexual minority individuals as a function of country-level structural stigma.

Despite accumulating evidence that structural stigma is strongly related to sexual minority individuals’ mental health, including by impacting individual-level stigma processes such as identity concealment and internalized stigma and emerging evidence that structural stigma is associated with an increased odds of victimization, no study has examined whether the association between structural stigma and any outcome might vary by diverse status characteristics within the sexual minority population. Two status characteristics in particular—gender nonconformity and lower socioeconomic status—are known to put some subgroups of sexual minority individuals at a particular risk of the negative impact of individual and interpersonal forms of stigma (Puckett et al., 2016; Thoma et al., 2021). Knowing whether subgroups of sexual minorities diverse along these status characteristics are also at greater risk of the downstream consequences of structural stigma can inform targeted supportive interventions across geographies, populations, and their intersection. The present study focuses on victimization as one possible downstream consequence to which these subgroups of sexual minorities might be disproportionately vulnerable.

In terms of gender nonconformity, sexual minorities who violate gender role norms are at increased risk of exposure to victimization. Recent meta-analytic evidence shows that gender nonconformity is consistently linked to experiencing more victimization, lower concealment of sexual orientation, and higher expectations of rejection (Puckett et al., 2016; Thoma et al., 2021). These studies specifically show that sexual minority men perceived as feminine and sexual minority women perceived as masculine are at particular risk of victimization (Lock, 2002). However, whether the risk of victimization toward gender nonconformity is greater in more structurally stigmatizing geographies remains unknown. Given that heterosexism in general is argued to be a manifestation of misogyny (Pharr, 1988), there is reason to believe that sexual minority men living in structurally stigmatizing countries might be at greatest risk of victimization given perceptions of sexual minority men as effeminate and in steeper violation of gender role norms compared to sexual minority women and heterosexual men (Herek, 2000).

Lower socioeconomic status, including low income or educational attainment, is also known to exacerbate the impact of stigma-related stress on outcomes such as poor mental health among sexual minority individuals. In general, sexual minority individuals with lower educational attainment are at greater risk of depression, anxiety, and substance use disorders (Barnes et al., 2014; McGarrity & Huebner, 2014). Fundamental cause theory (Phelan et al., 2004) stipulates that a lack of socioeconomic resources undermines health by making one vulnerable to the health-impairing mechanisms through which unfavorable structural conditions operate. Lower socioeconomic status is one such mechanism expected to place some sexual minority individuals at greater risk of the negative consequences of country-level conditions of structural stigma.

Intersectionality theory and research suggest that men and women’s experiences of victimization and violence are inseparably intertwined with other personal status characteristics, including gender nonconformity and socioeconomic status (Bowleg, 2012; Crenshaw, 1989; Gkiouleka et al., 2018). Experiences of victimization are elevated among both sexual minority men and women (Flores et al., 2020), but studies have also reported gender difference in the types of violence to which sexual minority individuals are exposed (Johns et al., 2020). For example, sexual minority young men in the U.S. are at higher risk for being threatened or injured with a weapon compared to sexual minority young women, whereas sexual minority young women are at higher risk for sexual violence (Johns et al., 2020). In general, studies show that sexual minority men are more likely to experience hate crimes and stigma-related experiences such as verbal harassment and discrimination (Herek, 2009; Herek et al., 1999). Sexual minority men are also more likely to be the target of mockery and rejection by peers and parents than sexual minority women owing to their gender nonconformity (Rieger & Savin-Williams, 2012; Young & Sweeting, 2004). Stigma-based rejection experienced by sexual minority men is particularly likely to emanate from other men possibly as a result of men’s greater negative attitudes toward gender nonconforming men versus gender nonconforming women (Herek, 2000). Violent victimization might be particularly likely to accrue to gender nonconformity among sexual minority individuals from lower socioeconomic backgrounds given the role of socioeconomic background as a fundamental cause of further marginalization among the already marginalized (Phelan et al., 2004). Intersectionality theory offers a framework for understanding how multiple intersecting identities linked to various social inequalities might be determined at a structural level. Indeed, intersectionality theory, as put forth by Black American feminists and legal scholars, ultimately locates the causes of disadvantage toward the multiply marginalized within structural factors based on, for example, racism, sexism, and heterosexism (Bowleg, 2012; Combahee River Collective, 1983; Crenshaw, 1989).

Based on the research and theory reviewed above, we hypothesized that: (a) sexual minority individuals living in higher structural stigma countries would report more victimization than those living in lower structural stigma countries and (b) the association between structural stigma and risk of exposure to victimization among sexual minority individuals would be strongest for those reporting gender nonconformity and lower socioeconomic status. As an exploratory intersectional hypothesis, we proposed that gender would further moderate the association among structural stigma, risk of exposure to victimization, and gender nonconformity and socioeconomic status.

## Method

### Participants

Data for the present study come from the European Union Lesbian, Gay, Bisexual, and Transgender (EU-LGBT) Survey (European Union Agency for Fundamental Rights, 2014). This online survey was administered between April and July 2012 by the European Union Agency for Fundamental Rights. The aim of the survey was to identify the fundamental rights affecting lesbian, gay, bisexual, and transgender (LGBT) individuals who were ≥18 years and lived in any of the 28 European Union member states at the time of the study (i.e., Austria, Belgium, Bulgaria, Croatia, Cyprus, Czech Republic, Denmark, Estonia, Finland, France, Germany, Greece, Hungary, Ireland, Italy, Latvia, Lithuania, Luxembourg, Malta, The Netherlands, Poland, Portugal, Romania, Slovakia, Slovenia, Spain, Sweden, and the United Kingdom). A multinational European team of sexual and gender minority experts developed the questionnaire, which was further translated into 27 languages using forward translation of an English-language version, followed by back translation. The final version of the questionnaire was verified for comprehension utilizing cognitive interviews conducted in five countries. The participants were recruited online via invitations posted to local, national, and international sexual and gender minority specific websites and through informal announcements about the survey posted primarily via Facebook pages and Twitter accounts belonging to national LGBT organizations. Interested individuals were directed to an online survey located on a secure server. The survey took about 28 minutes to complete (European Union Agency for Fundamental Rights, 2014).

In the present study, eligible respondents had to indicate understanding the study’s purpose and provide consent; reside in one of the 28 European Union countries at the time of the survey; be ≥18 years of age; and self-identify as lesbian, gay, or bisexual. The survey item assessing experiencing victimization was mandatory and assessed of all participants, so there was no missing data on this item. A final sample size of 86,308 sexual minority individuals were included in the analyses. The per-country range varied from 240 participants in Cyprus to 18,942 in Germany.

### Measures

#### Country-level measures

##### Country-level structural stigma

We calculated a score of structural stigma based on country legislation and population attitudes toward sexual minorities using a strategy similar to previous studies (Berg et al., 2013; Bränström et al., 2021; Pachankis & Bränström, 2018; Pachankis et al., 2015). Each country was assigned a score summarizing the anti-sexual minority structural stigma in that country in 2012. The score was calculated using a three-step procedure. First, we created an index of laws and policies toward sexual minorities collected by the International Lesbian, Gay, Bisexual, Trans and Intersex Association in Europe (ILGA-Europe, 2021). The index of laws and policies was created by calculating a sum score across the following six domains that identify different aspects of structural forms of discrimination and protection: (1) unequal age of consent for same-sex sexual acts, (2) presence of asylum provisions for sexual minority individuals, (3) protections against bias-motivated violence toward sexual minority individuals, (4) legal protections against discrimination toward sexual minority individuals, (5) same-sex partnership and parenting recognitions, and (6) freedom of assembly for sexual minority individuals. A country could be awarded up to 30 points for distinct supportive legal protections or lose up to 12 points for distinct types of discriminatory legislation. Hence, each form of protection was awarded a positive point whereas each form of discrimination was awarded with a negative point, yielding a scale that theoretically ranged from 30 to −12 (the actual range in this study was from 21 to 0). The scale was then reverse scored, so that a higher number indicated greater structural stigma. Second, we calculated an index of each country’s average attitudes toward sexual minority individuals utilizing an item from the European Social Survey (Norwegian Social Science Data Services, 2002–2018). Across all 28 countries, respondents to the European Social Survey were asked to rate their agreement with the statement: “Gay men and lesbians should be free to live their own life as they wish,” with the response options being “agree strongly”; “agree”; neither agree nor disagree”; “disagree”; and “disagree strongly.” The proportion of the population in each country disagreeing to the statement was calculated for each country (Norwegian Social Science Data Services, 2002–2018). In the final step, we standardized each measure and calculated the mean of the standardized policy index and the social attitude index to create a country-level index of structural stigma toward sexual minorities.

##### Country-level covariate

The Gini coefficient, an index of income inequality, was used as a country-level covariate, since income inequality has been linked with both increased prevalence of victimization (de Oliveira Ramos et al., 2017) and structural stigma toward sexual minority individuals (Andersen & Fetner, 2008).

#### Individual-level measures

##### Victimization

Frequency of exposure to *past-12-month victimization* was calculated using 2 items, namely (1) “In the last 5 years, have you been: physically/sexually attacked or threatened with violence at home or elsewhere (street, on public transport, at your workplace, etc.) for any reason?” with response options “yes” or “no” and (2) “When did the LAST physical/sexual attack or threat of violence happen?” with response options on this item “in the last 12 months” and “more than 12 months ago.” Those participants responding “yes” on the first item, and “In the last 12 months” on the second item, were categorized as having been exposed to victimization during the past 12 months.

##### Gender

Gender was coded based on responses to 2 items in the survey, 1 item concerning sex assigned at birth (i.e., “What sex were you assigned at birth?” With response options “female” and “male”) and 1 item concerning transgender experiences (i.e., “Are/were you a transgender person?” With response options “yes” and “no”). Participants reporting a transgender experience were subsequently asked to indicate their gender identity, but due to concerns of confidentiality and the small number of participants in some of these categories, the European Union Agency for Fundamental Rights did not permit access to the information about gender identity for the transgender participants. The analyses in the current study are therefore conducted only among cisgender participants. Participants were categorized as “men” if they were assigned male at birth and did not report a transgender experience. Participants were categorized as “women” if they were assigned female at birth and did not report a transgender experience.

##### Gender nonconformity

Participants were categorized as being *gender nonconforming* based on two survey questions, one concerning self-perceived felt femininity (i.e., “Do you agree or disagree with the following statements? I feel feminine” with the response options “strongly disagree,” “disagree,” “agree,” and “strongly agree”) and another concerning self-perceived felt masculinity (i.e., “Do you agree or disagree with the following statements? I feel masculine” with the response options “strongly disagree,” “disagree,” “agree,” and “strongly agree”). Participants categorized as “men” reporting agreement with feeling feminine (i.e., “strongly agree” or “agree”) or disagreement with feeling masculine (i.e., “strongly disagree” or “disagree”) were categorized as gender nonconforming. Participants categorized as “women” reporting agreeing to feeling masculine (i.e., “strongly agree” or “agree”) or disagreeing to feeling feminine (i.e., “strongly disagree” or “disagree”) were categorized as gender nonconforming. All other respondents were categorized as being gender conforming.

##### Socioeconomic status (SES)

Participants were classified into three levels of socioeconomic status (i.e., high, middle, and low) based on income and educational attainment. Educational attainment was coded into either high educational attainment (i.e., those with at least a university education) or low educational attainment (i.e., all educational levels below university degree). Income was coded based on self-reported annual household income into high (i.e., self-reported income higher than the median within the respective country) or low (i.e., self-reported income lower than the median within the respective country) income. Participants were coded as high SES if they reported high income and a university education, low SES if they reported low income and educational attainment below a university degree, and middle SES if they reported a combination of low and high educational attainment and income.

##### Sociodemographic covariates

Individual-level sociodemographic variables controlled for in this study included age, relationship status (i.e., single, in a same-sex relationship, or in an opposite-sex relationship), ethnic minority status (i.e., self-reported ethnic minority in current country of residence), and type of living area (i.e., urban vs. rural).

### Analytical Approach

In all analyses, we utilized multilevel modelling with individual-level factors (i.e., victimization, gender nonconformity, socioeconomic status, and sociodemographic covariates) modelled at Level 1 and country-level factors (i.e., structural stigma and Gini coefficient) modelled at Level 2. First, to examine whether country-level structural stigma predicted victimization, we conducted a generalized mixed effects model with a random intercept; effects were estimated using maximum likelihood parameter estimation. Second, to determine whether the association between country-level structural stigma and victimization was moderated by gender nonconformity and socioeconomic status, we employed multi-level moderation analyses with both random intercept and slopes. Multilevel moderation allowed us to test whether the association between each proposed moderator and victimization varied by country-level structural stigma. Third, we examined a model in which we explored whether gender would further moderate the interaction between structural stigma and gender nonconformity and socioeconomic status in predicting risk of exposure to victimization. The analyses were conducted using SPSS, version 26.

## Results

### Descriptive Statistics

Table 1 depicts the sociodemographic characteristics of the study sample. Most of the participants self-identified as gay or lesbian (84.2%) and were men (74.9%). Female participants were significantly younger than male participants (*p* < .001) and the majority of participants (89.0%) resided in an urban area. The standardized index of country-level structural stigma ranged from −1.46 in the United Kingdom to 2.08 in Latvia.

**Table 1.** Sociodemographics and Victimization for Participants in the EU-LGBT Survey 2012 by Gender.

		Gender	
	Total sample (*n* = 86,308)	Men (*n* = 64,648)	Women (*n* = 21,660)
	*n* (%)	*n* (%)	*n* (%)
Age			
18–29	53,774 (62.3)	37,417 (57.9)	16,357 (75.5)
30–39	17,706 (20.5)	14,457 (22.4)	3,249 (15.0)
40–49	10,579 (12.3)	9,049 (14.0)	1,530 (7.1)
50–59	3,321 (3.8)	2,898 (4.5)	423 (2.0)
60 or older	928 (1.1)	827 (1.3)	101 (0.5)
Sexual orientation			
Gay/lesbian	72,684 (84.2)	57,448 (88.9)	15,236 (70.3)
Bisexual	13,624 (15.8)	7,200 (11.1)	6,424 (29.7)
Ethnic minority status	6,073 (7.0)	4,717 (7.3)	1,356 (6.3)
Living area			
Urban	76,843 (89.0)	64,859 (89.2)	11,984 (88.0)
Rural	9,465 (11.0)	7,825 (10.8)	1,640 (12.0)
Relationship status			
Single	36,545 (42.3)	29,360 (45.4)	7,185 (33.2)
In a relationship, not living with a partner	23,747 (27.5)	16,718 (25.9)	7,029 (32.5)
Live with a partner	26,016 (30.1)	18,570 (28.7)	7,446 (34.4)
Gender nonconformity			
Gender conformity	77,867 (90.2)	60,905 (94.2)	16,962 (78.3)
Gender nonconformity	8,441 (9.8)	3,743 (5.8)	4,698 (21.7)
Socioeconomic status			
Low	24,326 (28.1)	17,997 (27.8)	6,329 (29.2)
Middle	35,794 (41.5)	26,263 (40.6)	9,531 (44.0)
High	26,188 (30.3)	20,388 (31.5)	5,800 (26.8)
Victimization			
Victimization, past 12 months	7,578 (8.8)	5,516 (8.5)	2,062 (9.5)

### Association Between Country-Level Structural Stigma and Victimization

**Figure 1.**
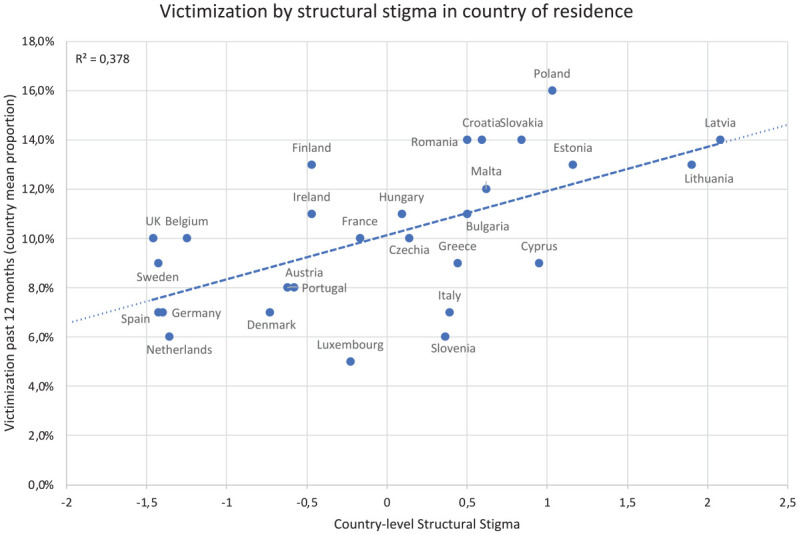
Past-12-month victimization among sexual minority individuals by country-level structural stigma in the EU-LGBT Survey 2012.

Results revealed a statistically significant association between country-level structural stigma and victimization after adjusting for covariates (adjusted odds ratios [AOR]: 1.13, 95% confidence intervals [CI]: 1.04, 1.22; *p* = .004), indicating that individuals living in countries with higher structural stigma were more likely to experience victimization than those living in countries with lower structural stigma. The proportion of sexual minorities exposed to victimization increased by 13% for each standard deviation increase in country-level structural stigma. Figure 1 presents average country-level proportion of exposure to victimization by country-level structural stigma.

### Moderation of the Association Between Country-Level Structural Stigma and Victimization by Gender Nonconformity and Socioeconomic Status

Multilevel moderation analyses showed no significant two-way interaction for structural stigma by gender nonconformity (*F* = 0.008, *p* = .928). However, there was a significant two-way interaction for structural stigma by socioeconomic status (*F* = 7.04, *p* = .008), showing that the association between structural stigma and experiences of victimization was positive and significant among sexual minorities with middle or low socioeconomic status (AOR: 1.18, 95% CI: 1.08, 1.28; *p* < .001) and nonsignificant among those with high socioeconomic status (AOR: 1.07, 95% CI: 0.95, 1.20; *p* = .252).

### Exploratory Moderation by Gender of the Association Between Country-Level Structural Stigma and Gender Nonconformity and Socioeconomic Status in Predicting Victimization

**Figure 2.**
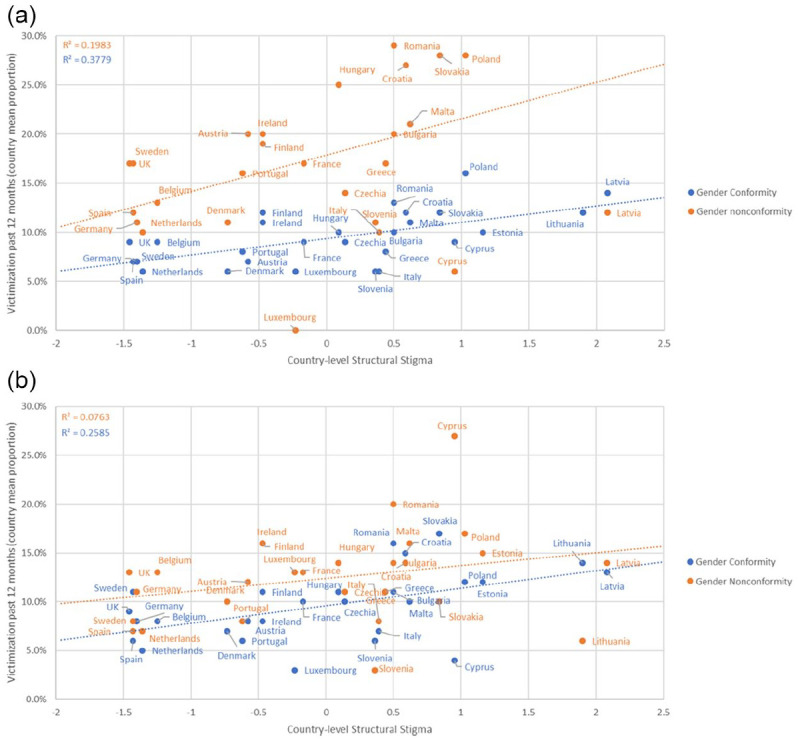
Past-12-month victimization by country-level structural stigma and gender nonconformity among: (a) sexual minority men and (b) sexual minority women, in the EU-LGBT Survey 2012.

The three-way interaction for Gender × Country-Level structural Stigma × Gender Nonconformity was significant in predicting past-12-month victimization (*F* = 4.21, *p* = .040). The interactions between country-level structural stigma and gender nonconformity stratified by gender are illustrated in Figure 2a and b.

**Figure 3.**
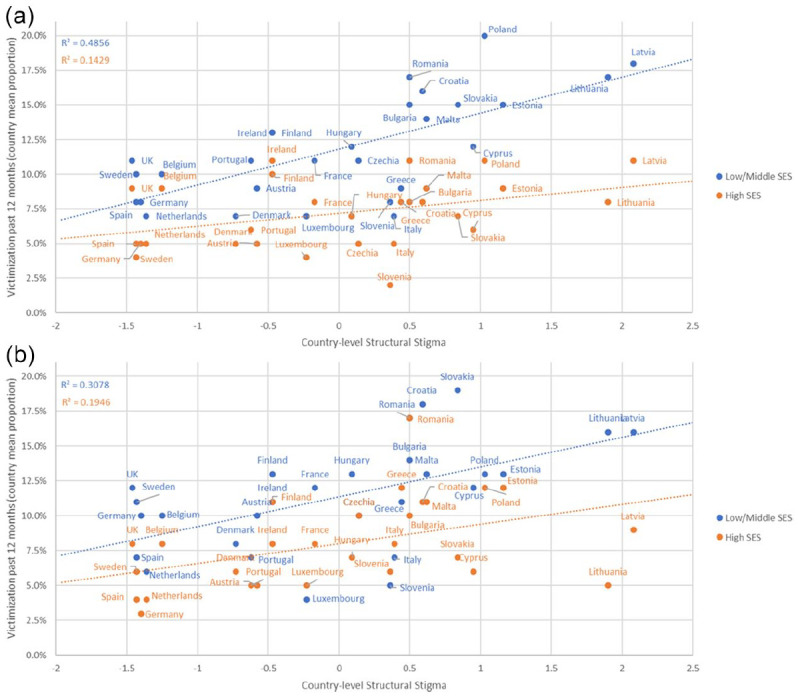
Past-12-month victimization by country-level structural stigma and socioeconomic status among: (a) sexual minority men and (b) sexual minority women, in the EU-LGBT Survey 2012.

The three-way interaction for Gender × Country-Level Structural Stigma × Socioeconomic Status was also significant (*F* = 7.16, *p* = .007). The interactions between country-level structural stigma and socioeconomic status stratified by gender are illustrated in Figure 3a and b.

The four-way interaction for Gender × Country-Level Structural Stigma × Gender Nonconformity × Socioeconomic Status was marginally significant (*F* = 2.81, *p* = .094), thereby motivating stratified analyses. Stratified analyses by gender showed that, among male participants, the main effect of gender nonconformity was significantly associated with victimization (AOR: 1.73, 95% CI: 1.62, 1.86; *p* < .001) and the main effect of socioeconomic status was significantly associated with victimization (AOR: 1.34, 95% CI: 1.25, 1.43; *p* < .001), demonstrating that victimization was more common among sexual minority men who reported gender nonconforming and low/middle socioeconomic status. Further, both the two-way interaction for structural stigma by gender nonconformity (*F* = 3.847, *p* = .049) and the two-way interaction for structural stigma by socioeconomic status (*F* = 7.626, *p* = .006) were significant. The association between structural stigma and victimization was stronger among gender nonconforming sexual minority men (AOR: 1.21, 95% CI: 1.05, 1.39; *p* = .009) compared to gender conforming men (AOR: 1.11, 95% CI: 1.03, 1.21; *p* = .008). The association between structural stigma and victimization was also stronger and significant among men with middle/low socioeconomic status (AOR: 1.18, 95% CI: 1.09, 1.28; *p* < .001) and nonsignificant among men with high socioeconomic status (AOR: 1.04, 95% CI: 0.92, 1.16; *p* = .558). Among men, the strongest association between structural stigma and victimization was found for gender nonconforming men with middle/low socioeconomic status (AOR: 1.32, 95% CI: 1.14, 1.52; *p* < .001).

Among female participants, gender nonconformity (AOR: 1.32, 95% CI: 1.20, 1.46; *p* < .001) and socioeconomic status (AOR: 1.29, 95% CI: 1.12, 1.49; *p* < .001) were significantly related to victimization with those reporting gender nonconformity and low/middle socioeconomic status having a higher risk of victimization. We found no significant two-way interaction for structural stigma by gender nonconformity (*F* = 3.388, *p* = .066) and no significant two-way interaction for structural stigma by socioeconomic status (*F* = 1.146, *p* = .284). However, there was a positive overall association between structural stigma and victimization among sexual minority women (AOR: 1.16, 95% CI: 1.03, 1.30; *p* = .010), showing that the proportion of sexual minority women exposed to victimization increased by 16% for each standard deviation increase in country-level structural stigma.

## Discussion

Although previous studies have found that sexual minority individuals are at disproportionate risk of victimization (D’Augelli et al., 2002; Fish et al., 2019; Friedman et al., 2011; Gordon & Meyer, 2007; Katz-Wise & Hyde, 2012), this study is among the first to show that differences in structural stigma toward sexual minorities across countries are systematically linked with increased risk of victimization toward this population. This study is also, to our knowledge, the first to explore a potential protective effect of gender conformity and socioeconomic status in the association between structural stigma and victimization risk and the first to demonstrate that such an effect was only present among gay and bisexual men. The present study utilized one of the largest datasets of sexual minority individuals to date and results showed that gender nonconformity and socioeconomic status functioned as significant effect modifiers of the association between country-level stigma and exposure to victimization. Gender-stratified analyses showed that the link between structural stigma and victimization was particularly strong among gender nonconforming men and among men with lower socioeconomic status, and the strongest association was found among gender nonconforming men with middle/low socioeconomic status. A moderating effect of gender nonconformity and socioeconomic status on the association between structural stigma and victimization was not found among sexual minority women.

The association between country-level structural stigma and victimization in this study is in line with one previous study focusing on sexual minority individuals across U.S. states, showing that individuals living in U.S. counties with fewer school districts with inclusive anti-bullying policies were more likely to experience peer victimization (Hatzenbuehler & Keyes, 2013). Thus, the current study extends our understanding of the impact of structural stigma and its impact on experiences of victimization among sexual minority individuals to a cross-country context, using a wider age range, and an objective measure of structural stigma based on country-level legislation and population attitudes.

Results showed that the structural context in which sexual minorities live influence the risk of victimization differently for those who are gender nonconforming and those who are gender conforming, and that this difference varies for sexual minority men and women. The strongest link between country-level structural stigma and victimization was found among gender nonconforming men, with sexual minority men living in high stigma countries are at greatest risk of victimization. Among women, the link between country-level structural stigma and victimization was similar among those who were gender conforming and those who were gender nonconforming, suggesting that the protective effect of gender conformity that exists among men does not seem to exist among women. However, gender conformity was protective against victimization among sexual minority women in all countries. To our knowledge, this is the first study to demonstrate how exposure to victimization in different structurally stigmatizing environments varies as a function of sexual minority men and women’s gender nonconformity. The greater risk of victimization among gender nonconforming men in high-stigma settings supports the possible link between structural stigma against sexual minorities and structural stigma against women, in that sexual minority men living in countries with high structural stigma toward sexual minorities are particularly likely to activate bias to the extent that they challenge gender norms and display feminine traits. The fact that we found such an effect for sexual minority men but not sexual minority women could be related to the fact that attitudes toward sexual minority men are generally more hostile than attitudes toward sexual minority women, especially when attitudes are measured among heterosexual men (Herek, 2000).

With respect to the protective effects of higher socioeconomic status, we found that among sexual minority men socioeconomic status moderated the association between country-level structural stigma and victimization, indicating that the association between structural stigma and risk of victimization is particularly strong among those with middle/low socioeconomic status. Although, we did not find a similar significant moderating pattern among sexual minority women, higher socioeconomic status was protective against victimization among sexual minority women in all countries. Although future studies are needed to investigate the reasons for this protective effect of socioeconomic status, it is possible that sexual minority individuals with higher socioeconomic status have greater access to safe environments, greater ability to choose their area of residence and occupation, and greater ability to decide with whom to socially interact, and thus have better access to non-stigmatizing resources within their countries of residence. These possibilities derive from the postulations of fundamental cause theory, which identifies the socially unequal distribution of knowledge, prestige, power, and supportive social resources as the basic causes of inequalities between advantaged and disadvantaged populations (Phelan et al., 2004). Although previous studies have indicated a protective effect of socioeconomic status on the mental health of sexual minority individuals (Barnes et al., 2014; McGarrity & Huebner, 2014), results of the present study extend this finding by showing that the association between living in a structurally stigmatizing context and risk of victimization depends on socioeconomic status. Most notably, the relatively lower risk of being victimized among European sexual minority men with high socioeconomic status does not seem to vary depending on their country of residence. This protective effect of higher socioeconomic status on the association between structural stigma and victimization was not statistically significant among sexual minority women.

These results should be interpreted in light of several study limitations. First, this study entails a cross-sectional study design, thus making it difficult to draw conclusions about causality. However, a reverse causal relationship is unlikely, given that victimization is unlikely to cause country-level structural stigma or be associated with migration patterns to shape such environments (Pachankis et al., 2021). Second, as is typical in population-based studies with sexual minority individuals, the EU-LGBT Survey is likely to underrepresent some members of this population, including those who are older, migrants, and not out (Hottes et al., 2016; Saewyc et al., 2004). Future studies utilizing probability-based sampling can allow broader generalization to the full population of sexual minority individuals. Third, since we did not have access to information about gender identity for transgender participants, we excluded transgender individuals from the analyses. This is particularly unfortunate given this population’s increased likelihood of being exposed to victimization (Blondeel et al., 2018). Future research with more detailed information about sex assigned at birth, transgender experiences, and gender identity is needed to better understand the impact of structural stigma on this population and the distribution of victimization based on gender nonconformity and socioeconomic status. Fourth, our assessment of victimization was limited to a measure of any past 12-month exposure to victimization. A more fine-grained and comprehensive assessment of different types of victimization would have been preferable given the known variety of victimization experiences reported in previous studies of sexual minority individuals (Scheer et al., 2020). Fifth, our assessment of gender nonconformity was limited to 2 items regarding felt masculinity and femininity. Yet gender nonconformity is a more complex construct that also includes behavioral expression and others’ impressions of one’s gendered presentation. Future research could ideally assess the full complexity of this construct using established guidelines (The GenIUSS Group, 2014). Finally, our assessment of structural stigma was limited to the country-level based on national legislation and population attitudes. Recent studies have indicated that more local/regional indicators of structurally stigmatizing environments are also important in predicting the experiences of sexual minority populations (Lattanner et al., 2021). Future research could benefit from assessing the structural environment at multiple levels, including national, regional, and community levels.

Overall, results of the present study suggest that the well-established risk of victimization toward sexual minority individuals is exacerbated in countries with higher structural stigma (Scheer et al., 2020; van der Star et al., 2021). These results extend existing research concerning the potential mechanisms of structural stigma to the interpersonal experience of victimization. Moreover, the present study extends existing research findings into the protective role of socioeconomic status and gender conformity by showing that these personal status characteristics also moderate the association between country-level structural stigma and victimization. In the present study, men in high structural stigma countries were most at risk of being victimized if they were gender nonconforming and if they had middle/low socioeconomic status.

Together, these findings support the core tenets of minority stress (Meyer, 2003) and fundamental causes theories (Phelan et al., 2004) by suggesting that the stress experiences of sexual minority individuals are determined by their broader structural environments. Findings also extend intersectional models of minoritized populations’ well-being by indicating that the experiences of intersectional social positions can be a function of structural stigma and its association with victimization directed toward the socially marginalized. The present findings suggest that social action to reduce stigmatizing national laws, policies, and attitudes related to a sexual minority status can be expected to lead to simultaneous reductions in sexual minority individuals’ exposure to victimization. Results also highlight subgroups within the sexual minority community, in particular sexual minority men who report gender nonconformity and lower socioeconomic status, who are at greatest risk of victimization in high-stigma environments and who the present results suggest might benefit the most from structural improvements.
